# Comparison of an alternative schedule of extended care contacts to a self-directed control: a randomized trial of weight loss maintenance

**DOI:** 10.1186/s12966-017-0564-1

**Published:** 2017-08-15

**Authors:** Gareth R. Dutton, Marissa A. Gowey, Fei Tan, Dali Zhou, Jamy Ard, Michael G. Perri, Cora E. Lewis

**Affiliations:** 1 Division of Preventive Medicine, Department of Medicine, University of Alabama at Birmingham, Birmingham, AL USA; 2 Department of Mathematical Sciences, Indiana University-Purdue University Indianapolis, Indianapolis, IN USA; 3 Department of Epidemiology and Prevention, Wake Forest University School of Medicine, Winston Salem, NC USA; 4 Department of Clinical and Health Psychology, University of Florida, Gainesville, FL USA

**Keywords:** Obesity, Behavioral treatment, Extended care, Weight loss maintenance, Weight regain, Adults

## Abstract

**Background:**

Behavioral interventions for obesity produce clinically meaningful weight loss, but weight regain following treatment is common. Extended care programs attenuate weight regain and improve weight loss maintenance. However, less is known about the most effective ways to deliver extended care, including contact schedules.

**Methods:**

We compared the 12-month weight regain of an extended care program utilizing a non-conventional, clustered campaign treatment schedule and a self-directed program among individuals who previously achieved ≥5% weight reductions. Participants (*N* = 108; mean age = 51.6 years; mean weight = 92.6 kg; 52% African American; 95% female) who achieved ≥5% weight loss during an initial 16-week behavioral obesity treatment were randomized into a 2-arm, 12-month extended care trial. A clustered campaign condition included 12 group-based visits delivered in three, 4-week clusters. A self-directed condition included provision of the same printed intervention materials but no additional treatment visits. The study was conducted in a U.S. academic medical center from 2011 to 2015.

**Results:**

Prior to randomization, participants lost an average of −7.55 ± 3.04 kg. Participants randomized to the 12-month clustered campaign program regained significantly less weight (0.35 ± 4.62 kg) than self-directed participants (2.40 ± 3.99 kg), which represented a significant between-group difference of 2.28 kg (*p* = 0.0154) after covariate adjustments. This corresponded to maintaining 87% and 64% of lost weight in the clustered campaign and self-directed conditions, respectively, which was a significant between-group difference of 29% maintenance of lost weight after covariate adjustments, *p* = 0.0396.

**Conclusions:**

In this initial test of a clustered campaign treatment schedule, this novel approach effectively promoted 12-month maintenance of lost weight. Future trials should directly compare the clustered campaigns with conventional (e.g., monthly) extended care schedules.

**Trial registration:**

Clinicaltrials.gov NCT02487121. Registered 06/26/2015 (retrospectively registered)

## Background

Behavioral weight management interventions typically produce initial reductions in body weight of 7–10%, which provides meaningful benefits for the prevention and management of various health conditions [1, 2]. Within the first year following treatment, however, individuals commonly regain 50–65% of their lost weight [3–5]. To attenuate this weight regain, extended care contacts are recommended for at least 1 year following initial treatment [1]. Indeed, extended care programs significantly reduce weight regain when compared to minimal or no additional contact [6–10]. By providing continued intervention contact focused on maintaining weight loss, participants are assisted in sustaining the self-management strategies necessary for long-term weight management [5, 7, 8].

Despite the documented benefits of maintenance programs, less is known about the best schedule for delivering these programs. Weight loss maintenance programs generally provide contacts on an evenly-spaced schedule, such as bi-weekly or monthly visits [1, 11, 12]. According to learning theory and theories of behavior change [13–15], this fixed-interval schedule is appropriate for the *initiation* of treatment and learning of new behaviors. This intensive contact schedule provides ongoing opportunities to learn and practice self-management skills while receiving support and corrective feedback from trained interventionists [5, 16].

However, as individuals transition to maintaining self-management behaviors in the extended care phase of behavioral obesity treatment, it is unclear whether the same schedule or intensity of contact is appropriate. In fact, operant learning, self-regulation, and relapse prevention theories [5, 6, 13–20], combined with preliminary research, suggest that an alternative schedule of treatment delivery may be useful for behavior *maintenance* [21, 22]. More specifically, a temporally-clustered campaign includes periods of frequent contact separated by extended periods without contact, which provides opportunities for intensive support and expert feedback over a shorter time period to further develop skills for weight maintenance behaviors; yet, it also allows for independent practice of self-management skills for extended periods.

Additionally, a clustered campaign schedule during the maintenance phase of treatment may protect against diminished reinforcement, boredom, and behavioral habituation that are common with conventional maintenance schedules [5, 16, 23–26]. Such clustered campaigns can be organized around different themes that may further reduce monotony, which is not typically used in the initial phase of behavioral obesity treatment when novel content is being introduced regularly. It also allows advanced knowledge acquisition and honing of advanced skills during maintenance. Variability in the schedule and content may counter the boredom that can occur during the sessions (e.g., participants get a ‘break’ from group participation for several weeks between campaigns that might in turn improve engagement when returning to group) as well as habituation with engaging in the same routine and behaviors between sessions. Despite the potential benefits, temporally-clustered sessions may increase the risk of attrition and less timely support due to extended periods without contact.

Contemporary weight loss programs, such as the Look AHEAD trial and Diabetes Prevention Program [27, 28], have incorporated “refresher groups” or “motivational campaigns” delivered on this temporally-clustered type of schedule during the maintenance phases of treatment. However, this alternative maintenance schedule was one part of a multi-component intervention that included continuation of evenly-distributed, fixed-interval contacts. Two other trials also used temporally-clustered extended care contacts to reduce weight regain [21, 29], although novel scheduling was one of several simultaneous treatment modifications, including different intensities of contact [29] and different behavioral prescriptions and goals [21].

Thus, the specific effect of temporally-clustered schedules for extended care remains unclear. To provide an initial test of this alternative extended care schedule in the absence of other treatment components, the purpose of this behavioral extended care trial was to compare 12-month outcomes of a clustered campaign maintenance delivery schedule to a self-directed comparison condition among individuals who previously achieved ≥5% weight reductions. It was hypothesized that an extended care program that included 12 maintenance visits delivered in three, 4-week clusters, would result in significantly better maintenance of lost weight than the comparison condition.

## Methods

### Participants

Participants were adults (≥21 years-old) who had taken part in a 16-week weight loss program and lost ≥5% of their initial body weight by month 4 of the program. Inclusion criteria for the initial, 16-week program included a body mass index (BMI; kg/m^2^) between 28 and 45. Individuals were ineligible to participate in the initial program if they had lost >4.5 kg and/or taken a weight loss medication in the past 6 months, had a medical condition for which weight loss or physical activity would be inadvisable, planned to relocate from the area in the next 18 months, were unable or unwilling to attend sessions, or were unwilling to accept random assignment. Participants were recruited through local newspaper, television, flyer, and university-affiliated website and e-newsletter advertisements.

### Procedures

**Fig. 1:**
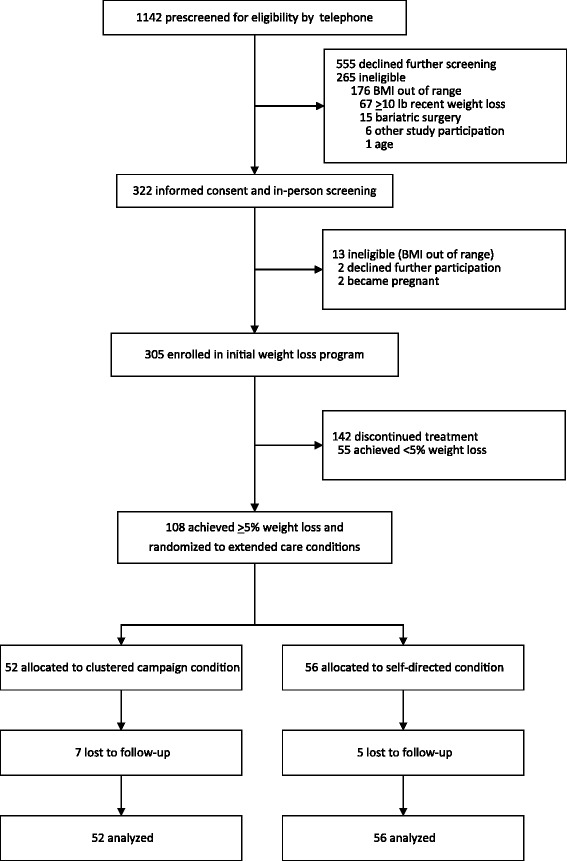
CONSORT flow diagram

Interested individuals contacted project staff to complete a pre-screening interview by telephone to determine preliminary eligibility. Based on the telephone screening, eligible individuals were scheduled to attend an orientation session and provide informed consent. This included brief, in-person screening to confirm eligibility (i.e., measurement of height and weight for BMI). Participant flow is summarized in Fig. 1. Participants providing consent returned for a baseline assessment visit and enrolled in an initial 16-week weight loss program.

This initial program included weekly, group-based sessions at an academic medical center with 15–20 participants. Groups were facilitated by a team of trained interventionists, including dieticians, exercise specialists, and clinical psychologists. Program content was modeled after the Diabetes Prevention Program (DPP) [28] and weight loss protocols by Perri and colleagues [7]. Participants were encouraged to work toward a 5% weight loss goal, and they were aware this criterion was required to continue into the maintenance trial. They were instructed to reduce caloric intake to 1200 kcal/day (for participants weighing <250 lb) or 1500 kcal/day (for participants weighing ≥250 lb) and encouraged to work toward a physical activity goal of at least 180 min/week. Participants received training in behavioral strategies designed to support weight loss, including goal-setting, problem-solving, and self-monitoring. Participants also received pedometers, food scales, and measuring cups/spoons to facilitate self-monitoring. Although the initial program provided intensive (i.e., weekly) contact and evidence-based content, treatment did not include an extensive behavioral run-in period, individual make-up sessions for missed groups, or other supplemental resources or strategies to maximize adherence and weight loss as provided in prior programs such as DPP and other weight loss trials.

Upon completion of the initial program, participants attended an assessment visit and those who achieved ≥5% weight loss were randomized in a parallel 1:1 ratio to one of two maintenance conditions for a 12-month extended care trial. At this visit, staff provided eligible participants with a numbered, sealed envelope, which indicated to which condition they were randomized. Randomization was prepared by PROC PLAN in SAS (Ver. 9.3), and a unique randomization number was generated for each participant. A permuted-block randomization scheme with block sizes of 4 was used to maintain assignment balance. Approval for this study was obtained from the IRB of the participating academic health center. Informed consent was obtained from all individual participants included in the study. This trial is registered with clinicaltrials.gov (NCT02487121).

### Extended care interventions

#### Clustered campaign maintenance program

Consistent with other extended care programs [7, 30], the purpose was to maintain adherence, bolster motivation, and reinforce information previously discussed with a focus on the maintenance of healthy lifestyle behaviors. Sessions included some review of previously-learned concepts and skills as well as incorporation of novel material pertaining to diet, physical activity, and behavior/motivation. The main focus of the extended care sessions was on the maintenance (rather than initial adoption) of healthy lifestyle behaviors associated with continued weight loss and/or weight loss maintenance and the avoidance of returning to less healthy behaviors.

The unique feature of this maintenance program was the non-conventional schedule of delivery. Rather than an evenly-distributed, fixed-interval (e.g., monthly) schedule of contact, the program included 12 extended care group sessions divided into three intensive, 4-week campaigns. These occurred during months 7, 10, and 13 of the 16-month study (Table 1). Thus, the periodic episodes of frequent visits were separated by extended periods without contact. Session content of the three campaigns was organized to reflect themes, including overviews of dietary practices (sessions 1–4), physical activity (sessions 5–8), and behavioral and motivational strategies and relapse prevention (sessions 9–12). This schedule of several weekly meetings separated by extended periods with less frequent contact is consistent with the campaigns or refresher groups offered in the Look AHEAD trial during extended care [27].

**Table 1:** Clustered campaign intervention schedule for weight loss maintenance

Month	1	2	3	4	5	6	7^a^	8	9	10^b^	11	12	13^c^	14	15	16
Treatment Contacts	X X X X	X X X X	X X X X	X X X X			X X X X			X X X X			X X X X			
	Initial intervention (16 contacts in months 1-4)				Clustered campaign extended care intervention (12 contacts in months 5-16)											

X = Group-based treatment contact; ^a^ Dietary campaign; ^b^ Physical activity campaign; ^c^ Behavioral strategies campaign

During extended care, participants established weight goals mutually agreed upon by them and interventionists. Goals could include additional weight loss or weight maintenance, depending on individuals’ progress, desired weight loss, and current BMI. Across all three campaigns, recommended behavioral goals for each participant included dietary and activity self-monitoring at least 3 days/week. In addition to self-monitoring goals, participants were encouraged to set campaign-specific dietary, physical activity, or behavioral/relapse prevention goals corresponding to specific session content (e.g., applying volumetric techniques to modify meals, identifying a new structured exercise to implement). Although general goals were provided to participants at each session, participants were encouraged to develop individualized action plans to tailor and implement each goal.

Because the trial included only participants losing ≥5% of baseline weight, fewer groups were required to deliver extended care treatment than during the initial weight reduction program. Therefore, extended care participants were consolidated into new treatment groups at randomization, which included a combination of familiar and new group members. The same team of interventionists delivered the initial and extended care programs. To ensure fidelity to treatment delivery, all interventionists participated in initial training on the intervention protocol, which included structured facilitator guides and participant treatment materials. Although treatment fidelity was not formally assessed, all interventionists participated in ongoing weekly group supervision directed by the study PI, a licensed clinical psychologist with extensive experience in the delivery of behavioral weight management programs. Content of these group discussions indicated consistent protocol adherence.

#### Self-directed program

Participants in the self-directed program received printed intervention materials at the initiation of the 12-month extended care period. These materials were identical to those provided in the clustered campaign condition. Self-directed participants initially met with an interventionist to review the treatment materials and answer any questions about the extended care program. They were encouraged to continue using behavioral strategies for weight management including self-monitoring and independently work through the written materials provided. The self-directed control group did not receive further in-person contacts with interventionists during the 12-month follow-up. If participants initiated contact with interventionists by telephone or email during the extended care period, the interventionist provided brief feedback, responded to questions or concerns, and referred participants back to the intervention materials provided at randomization.

### Measures

#### Demographics and medical history

At baseline, participants self-reported a variety of characteristics, including age, sex, race, educational attainment, income, and marital status.

#### Anthropometric measures

Height was measured at baseline to the nearest 0.1 cm using a wall-mounted stadiometer. Weight was measured to the nearest 0.1 kg using a calibrated digital scale at three time points, including baseline (i.e., pre-treatment), month 4 (i.e., randomization), and month 16. The experimental sample included those participants who lost ≥5% body weight by month 4 and were randomized into the 12-month trial. The primary outcome was change in body weight during the 12-month randomized trial, which occurred between months 4 and 16. Secondary outcomes included the proportion of participants in each extended care condition that maintained ≥5% and ≥7% weight reductions at month 16. These values were selected because 5% weight loss is considered a clinically significant value for chronic disease risk reduction [1], greater weight loss (e.g., 7%) is associated with more pronounced health benefits [1, 31], and 7% weight loss is consistent with the goals of other lifestyle interventions such as Look AHEAD and the Diabetes Prevention Program [27, 28]. Height and weight were measured in a research clinic affiliated with the academic medical center by trained assessment staff with no involvement in initial or extended care treatment delivery.

### Statistical analyses

A priori calculations indicated that *N* = 96 was required to provide statistical power of 80% to detect a between-group difference of 2.6 kg with a standard deviation of 4.5 kg at month 16. Accounting for 10% attrition, the required sample was 106, which was exceeded in this trial (*N* = 108). Based on previous trials [32, 33], it was conservatively estimated that one-third of participants would achieve ≥5% weight loss. Thus, to achieve 106 randomized participants, we estimated that 318 participants would need to be enrolled initially.

To summarize sample characteristics, descriptive statistics (sample mean, proportions and standard deviations) are provided. To analyze the effect of the clustered campaign maintenance program on the primary outcome of weight change over the extended care period (month 4 to month 16), two multiple regression models were fitted with response variables being weight change (kg) and weight regain (percent). In both models, the variables accounted for included age, marital status, race, education level, weight at randomization, and initial weight change (between months 0 and 4). To analyze the secondary outcome of the likelihood of maintaining a certain percentage of weight reduction at month 16, we dichotomized the weight loss percentage from month 0 to month 16 at ≥5% and ≥7% reductions from month 0. Logistic regression model was applied to each outcome. In addition to the treatment variable, we also accounted for baseline weight at month 0, age, marital status, race, and education. Odds ratio estimates and *p*-values were obtained.

As summarized in Fig. 1, 12 of 108 randomized participants were missing weight values at month 16 (i.e., 11% attrition). Attrition did not differ between conditions, *p* = 0.45. Due to these missing follow-up weight data and based on current recommendations for handling such data in weight loss trials [34], primary and secondary analyses were conducted based on multiple imputation in which we imputed the missing month 16 values 100 times to create 100 complete data sets, applied the desired analysis model to every imputed data set, and then pooled parameter estimates from the 100 data sets to form the final model results. Available variables used as predictors of missing data included treatment condition, race, marital status, education, age, income, height, initial weight (kg), and randomization weight (kg). Sensitivity analyses also were conducted with complete cases only, which yielded comparable results to those including multiple imputations. Thus, only the full sample intent-to-treat results with multiple imputation of missing data are presented here. Statistical analyses were implemented in R software [35] and SAS software, Version 9.4 of the SAS System for Windows.

## Results

### Participant characteristics

Recruitment, treatment delivery, and data collection were ongoing for this project from 11/01/11 to 3/31/15. Baseline (month 0) characteristics of the randomized sample are summarized in Table 2. The sample was comprised primarily of women (*n* = 103; 95.4%) and racial/ethnic minorities (*n* = 59; 54.6%). Of the 59 minority participants, most self-identified as African American (*n* = 56) followed by Asian (*n* = 1) and “other” racial group (*n* = 2).

**Table 2:** Baseline characteristics of randomized participants ^a^

Characteristic ^a^	Clustered Campaign Group (*n* = 52)	Self-directed Group (*n* = 56)	Total Randomized Sample (*N* = 108)
Age, years	52.13 (11.75)	51.18 (14.22)	51.64 (13.03)
Gender, No. (%)			
Male	1 (1.92)	4 (7.14)	5 (4.63)
Female	51 (98.08)	52 (92.86)	103 (95.37)
Race, No. (%)			
Non-Caucasian	28 (53.85)	31 (55.36)	59 (54.63)
Caucasian	24 (46.15)	25 (44.64)	49 (45.37)
Marital status, No. (%)			
Not Married	20 (38.46)	30 (53.57)	50 (46.30)
Married	32 (61.54)	26 (46.43)	58 (53.70)
Education, No. (%)			
Associates or lower degree	17 (32.69)	23 (41.07)	40 (37.04)
Bachelors or higher degree	35 (67.31)	33 (58.93)	68 (62.96)
Income, No. (%)			
≤ $40,000	13 (25.00)	19 (33.93)	32 (29.63)
$40,000 - $80,000	23 (44.23)	21 (37.50)	44 (40.74)
≥ $80,000	16 (30.77)	16 (28.57)	32 (29.63)
Body weight, kg	92.44 (13.77)	92.69 (12.86)	92.57 (13.24)
BMI, kg/m^2^	34.93 (4.04)	35.26 (4.21)	35.10 (4.11)

*Abbreviations*: *BMI* body mass index

^a^Data are given as mean (SD) unless otherwise indicated. All values are at the start of the initial weight loss program (i.e., month 0)

### Initial weight loss (months 0 to 4)

While 305 participants initially enrolled in the pre-randomization weight loss program, 108 (35.4% of original sample) completed the initial program and achieved the specified criterion of ≥5% weight loss. Therefore, 108 participants were randomized into the 12-month extended care trial (Fig. 1). All subsequent analyses are based on the randomized, extended care sample. Table 3 summarizes the pre-randomization weight change of these participants during the initial 16-week program, which represented significant within-group reductions in body weight (mean [SD], −7.45 [3.11] kg in the clustered campaign group and −7.64 [2.99] kg in the self-directed group).

**Table 3:** Changes in weight during the initial 4-month weight loss program (Months 0 to 4) ^a^

Outcome	Clustered Campaign Group (*n* = 52)	Self-directed Group (*n* = 56)	Total Randomized Sample (*N* = 108)
Body weight, kg	−7.45 (3.11)	−7.64 (2.99)	−7.55 (3.04)
Body weight, %	−8.05 (3.01)	−8.22 (2.83)	−8.14 (2.91)
BMI, kg/m^2^	−2.86 (1.17)	−3.03 (1.13)	−2.95 (1.15)

*Abbreviations*: *BMI* body mass index

^a^Data are given as mean (SD)

### Weight loss maintenance (months 4 to 16)

**Fig. 2:**
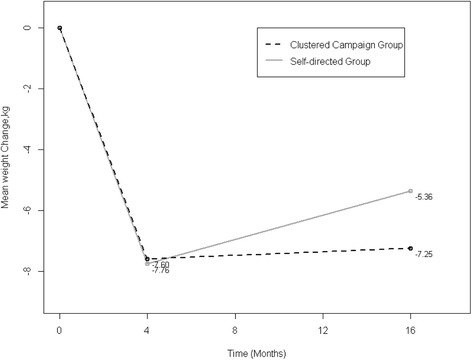
Mean weight change by extended care condition. Randomization occurred at month 4. Reported mean weight change includes unadjusted, observed means for 96 randomized participants who completed the follow-up assessment at month 16. Multiple linear regression indicated significantly less weight regain in the clustered campaign group vs. the self-directed group between months 4 and 16 (*p* = 0.0154)

Participants randomized to receive the clustered campaign program regained less weight (unadjusted mean [SD], 0.35 [4.62] kg) compared to those in the self-directed condition (unadjusted mean [SD], 2.40 [3.99] kg) over the 12-month extended care period (Fig. 2). After adjustment for covariates in the multiple regression model, this represented a significant between-group difference in weight regain of 2.28 kg, *P*-value = 0.0154, favoring the clustered campaign program (Table 4). Similar results were obtained when the outcome was proportion of weight regain. These values equated to clustered campaign participants regaining 13% (unadjusted mean [SD], 13.11 [57.51] percent) of their initial weight loss, which compared favorably to the nearly 36% regain (unadjusted mean [SD], 35.55 [61.85] percent) observed for self-directed participants. After adjustment for relevant covariates, this represented a significant between-group difference of nearly 29% greater regain in the self-directed condition, *P*-value = 0.0396 (Table 5).

**Table 4:** Linear regression analysis of weight change (kg) during the 12-month extended care program (Months 4 to 16) ^a^

Independent Variable ^b^	Weight Change, kg		
	Parameter Est.	Std. Err	*P*-value
Treatment Condition	−2.28	0.92	0.0154
Randomization Weight	−0.02	0.04	0.6193
Initial Weight Change	0.26	0.15	0.0779
Age	<0.01	0.04	0.9197
Race	−0.73	1.08	0.5015
Marital Status	−1.33	0.94	0.1589
Education	−0.62	0.95	0.5124

^a^Analyses are based on multiple imputation of missing data

^b^For categorical independent variables, the comparison level versus reference level are as follows: Treatment Condition (Clustered Campaign vs. Self-directed), Race (Non-Caucasian vs. Caucasian), Marital Status (Not-married vs. Married), Education (Bachelors or higher degree vs. Associates or lower degree)

**Table 5:** Linear regression analysis of percentage weight regain during the 12-month extended care program (Months 4 to 16) ^a^

Independent Variable ^b^	Weight Change, %		
	Parameter Est.	Std. Err	*P*-value
Treatment Condition	0.29	0.14	0.0396
Randomization Weight	<0.01	0.01	0.4582
Initial Weight Change	−0.06	0.02	0.0170
Age	<0.01	0.01	0.9578
Race	0.04	0.17	0.8106
Marital Status	0.21	0.14	0.1409
Education	0.10	0.14	0.4830

^a^Analyses are based on multiple imputation of missing data

^b^For categorical independent variables, the comparison level versus reference level are as follows: Treatment Condition (Clustered Campaign vs. Self-directed), Race (Non-Caucasian vs. Caucasian), Marital Status (Not-married vs. Married), Education (Bachelors or higher degree vs. Associates or lower degree)

Secondary analyses compared the proportions of participants maintaining a reduced body weight that was at least 5% and 7% below initial (month 0) levels upon completion of the 12-month extended care program at month 16. A slightly greater proportion of clustered campaign participants (62.2%) maintained ≥5% weight reductions compared to self-directed participants (54.9%), although this was not significantly different, *p* = 0.47. However, the proportion achieving ≥7% reductions differed significantly between clustered campaign (51.1%) and self-directed (29.4%) conditions, *p* = 0.03. The adjusted likelihood of achieving ≥5% and ≥7% weight loss at the final follow-up was 1.45 and 2.79 times greater for clustered campaign participants as compared to self-directed participants, *p’*s = 0.3959 and 0.0283, respectively (Table 6).

**Table 6:** Likelihood of achieving ≥ 5% and ≥ 7% weight loss maintenance at month 16 in the clustered campaign versus self-directed condition^a^

Weight Loss	Odds Ratio Estimate^b^	95% Confidence Interval	*P*-value
≥5%	1.45	0.62–3.41	0.3959
≥7%	2.79	1.12–6.98	0.0283

^a^Analyses are based on multiple imputation of missing data

^b^Odds ratio estimates and *P*-values for treatment effect (Clustered campaign vs. Self-directed) were obtained from logistic regressions. Variables accounted for in each logistic model included: baseline weight at month 0, age, race, marital status and education. None of these variables was significant

## Discussion

While previous trials have incorporated clustered campaign schedules into extended care programs to improve long-term weight management, this approach has been included along with other treatment components [27, 28] and/or additional alterations in delivery [21, 22, 29]. Thus, our understanding remains limited about the effect of using only a clustered campaign schedule to prevent weight regain, so the purpose of this study was to conduct an initial evaluation of such a schedule without other treatment components or adaptations (e.g., ongoing fixed-interval contacts, changes in dietary or behavioral targets). In the current sample of treatment-seeking, racially-diverse adults, a clustered campaign extended care program without major additional treatment components significantly improved maintenance of lost weight compared to a self-directed condition. The clustered campaign program resulted in the regain of only 13% of initial weight losses. Individuals in the self-directed condition regained nearly 29% more weight on average.

These findings are encouraging, since the first year following treatment represents a particularly high-risk period for weight regain. During this time, documented levels of weight regain are as high as 65%, which often precedes full weight regain within 3–5 years after treatment [4]. While comparisons to other weight loss maintenance trials are complicated by differences in aspects of treatment, populations, and settings investigated, the current effects appear consistent with outcomes achieved in conventional fixed-interval interventions [4, 6, 7, 30, 36]. Across a number of trials, fixed-interval sessions provided after the first 6 months of treatment resulted in the maintenance of approximately 66% of lost weight [4].

In the intensive lifestyle intervention of the Look AHEAD trial, which included periodic campaigns during maintenance in conjunction with other treatment components, weight losses were 8.5%, 6.3%, and 4.7% at 12-, 24-, and 48-months post-randomization, respectively [37]. Direct comparisons between Look AHEAD and the current trial are complicated by differing study designs (i.e., randomization occurring at baseline versus after initial weight reduction), different lengths of follow-up, and different target populations (i.e., adults with type 2 diabetes in Look AHEAD). However, the current 7–8 kg weight loss observed at month 4 and the maintenance of >7 kg weight loss observed at month 16 (i.e., 12 months post-randomization) are generally consistent with the first 2 years of follow-up in Look AHEAD.

The other trial most similar to the current one involved delivery of six, 8-week units of sessions that were separated by 4 weeks without contact [21]. While this novel timing of contacts seemed beneficial for weight loss maintenance in particular [21, 22], this alternative intervention included a number of other modifications that potentially impacted outcomes, including variety in the behavioral prescriptions, foci, and homework assignments of each unit as well as the use of structured financial incentives/contracts in one unit. Together, findings from the current trial and those observed by Jeffery, Levy, and colleagues [21, 22] support the potential value of a clustered campaign schedule of contacts to promote the maintenance of lost weight and highlight the need for additional research to more fully examine this approach.

### Strengths and limitations

The study design and protocol adhered to a structured, intensive, evidence-based program consistent with current treatment guidelines [1]. Randomizing participants to maintenance and comparator conditions after completion of an initial program and achievement of a pre-specified weight loss criterion is consistent with current methodological recommendations [38] and other trials of weight loss maintenance [6, 7, 36]. In contrast, some trials randomize all participants at the beginning of treatment, which is appropriate for understanding overall, long-term weight change rather than weight loss maintenance in particular [38]. Each design has strengths and limitations, and randomizing a subset of the original sample may limit generalizability of findings to the broader population of individuals seeking weight loss treatment. In the current study, for example, only 35% of initially enrolled participants lost ≥5% to become eligible for randomization. In addition, only 53% of the original (pre-randomization) sample completed the initial, 4-month weight loss program. Although this protocol included evidence-based levels of contact during initial treatment, it provided minimal additional resources, make-up sessions, or other efforts to bolster attendance or adherence for those who were not initially responding to treatment. In addition, there was limited pre-screening of individuals and relatively modest exclusion criteria for enrollment, which contrasts with extensive screening procedures and behavioral run-in periods common to other weight loss trials (e.g., DPP, Look AHEAD).

The racially-balanced sample was a strength, as well as the high retention rate (89%) at follow-up. However, as 95% of participants were women, findings may not generalize to men. Furthermore, there were a high proportion of African American women, a group demonstrating more modest weight loss during the first few months of lifestyle interventions [39–41]. The current treatment response (i.e., 35% achieved pre-specified criterion of success) is lower than the 61% of participants who achieved a > 4-kg loss in the similarly-designed Weight Loss Maintenance (WLM) trial [36]. Compared to the WLM trial, however, the current trial included a higher proportion of African American women (51% vs. 32%), a shorter duration of initial treatment (16 vs. 20 weekly sessions), and a slightly different weight loss criterion (5% vs. 4 kg) [40].

Although the clustered campaigns may promote novelty and offer an alternative approach to a conventional fixed-interval schedule, the experimental treatment schedule is most accurately conceptualized as a modified, fixed-interval schedule. This schedule did not include unpredictable, variable contacts, and other schedules of contact may prove superior to the one examined here [13–15]. Also, the self-directed control condition does not provide a direct comparison of the clustered campaign to a conventional, fixed-interval schedule that is comparable in treatment intensity. Given the limited literature on differing contact schedules for extended care programs, this was designed as an initial test of a clustered campaign maintenance schedule isolated from the effects of other treatment components. Importantly, this initial test included a randomized design, which offers a more rigorous approach than a quasi-experimental or single-group study design. Thus, the current findings are a novel, methodologically-sound addition to the literature that offers a promising approach to promote weight loss maintenance that requires replication and further exploration. Furthermore, other behavioral and/or psychological changes that were not measured may have occurred during treatment and could potentially mediate or moderate the effects observed for weight loss. Finally, while the duration of extended care is consistent with other maintenance trials [4, 7, 9, 30, 42], it is unknown what would occur with the maintenance of lost weight beyond the 12-month extended care period.

### Future research

This trial provides a promising first step in understanding the effects of an alternative schedule of contacts for extended care weight management programs. Future research directly comparing clustered campaign and fixed-interval schedules is needed to determine whether alternative extended care schedules minimize weight regain relative to conventional schedules of continued contact. Moreover, the examination of potential moderators and/or mediators of treatment effects are needed to understand why certain maintenance schedules may be advantageous. Alternative methods of delivering clustered campaign extended care treatment should be considered, including other schedules of contact and modalities of delivering treatment. The success with the clustered campaign approach for maintenance of weight loss in the current study raises the question of whether this approach may also be worthwhile for initial weight management treatment. Similarly, it may be useful to explore this approach for the maintenance of other behavioral changes prone to relapse that adhere to similar structured programs (e.g., smoking cessation).

## Conclusions

In summary, these findings demonstrated that individuals who participated in an extended care intervention with a novel, clustered campaign delivery approach had better weight maintenance following an intensive lifestyle intervention than those who participated in a self-directed maintenance condition. Thus, extended care interventions for sustained weight loss may be efficaciously delivered via clustered campaign schedules or other alternative treatment schedules. Further research is needed to test these novel delivery methods against more conventional extended care delivery approaches to determine whether they have added weight maintenance or cost benefits.
